# Cerebrotendinous Xanthomatosis With a Heterozygous Frameshift Mutation Involving *CYP27A1(C.526del)*

**DOI:** 10.1210/jcemcr/luaf153

**Published:** 2025-07-16

**Authors:** Ajitesh Roy, Sounak Kumar Roy, Soumyadip Das

**Affiliations:** Department of Endocrinology, Vivekananda Institute of Medical Sciences, Kolkata, West Bengal 700026, India; Department of General Medicine, Vivekananda Institute of Medical Sciences, Kolkata, West Bengal 700026, India; Department of General Medicine, Vivekananda Institute of Medical Sciences, Kolkata, West Bengal 700026, India

**Keywords:** cerebrotendinous xanthomatosis, CYP27A1, c.526del, xanthoma

## Image Legend

**Figure 1.**
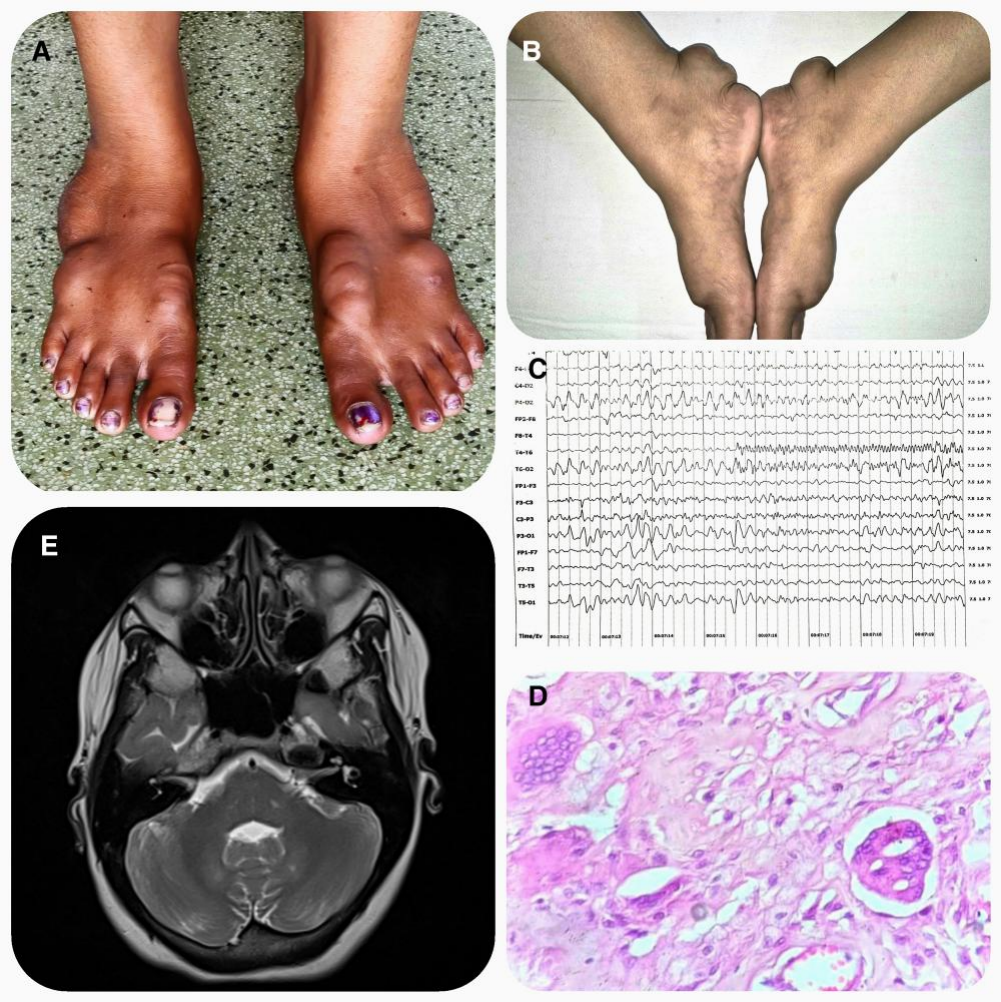
(A) Multiple tendon xanthomas. (B) Tendon xanthomas involving Achilles tendon. (C) Electroencephalogram showing generalized sharp wave discharges in both hemisphere. (D) Tendon xanthoma histopathological examination showing foamy histiocytes and Touton giant cells. (E) T2 weighted magnetic resonance imaging showing hyperintensities in the bilateral dentate nuclei.

A 31-year-old female presented with painless, progressive focal swelling in both feet over the past 4 years. There was no history suggestive of any cardiac or neurologic abnormalities. On examination, multiple lumps were observed in each foot, including over the Achilles tendon, which were soft and nontender (Fig. 1A and 1B). Neurologic and cardiac examinations revealed no abnormalities. Investigations showed subtle dyslipidemia [cholesterol: 207 mg/dL (5.36 mmol/L) (normal reference range: less than 200 mg/dL; 5.17 mmol/L); triglycerides: 351 mg/dL (3.96 mmol/L) (normal reference range: less than 150 mg/dL; 1.7 mmol/L); low-density lipoprotein: 103 mg/dL (2.67 mmol/L) (normal reference range:100 mg/dL; 2.6 mmol/L)]. Electroencephalogram revealed generalized sharp wave discharges (Fig. 1C). Histopathological examination revealed foamy histiocytes and Touton giant cells (Fig. 1D). Magnetic resonance imaging demonstrated T2 hyperintensities in the bilateral dentate nuclei (Fig. 1E). Exome sequencing showed a pathogenic heterozygous frameshift mutation involving c.*526del* in exon 3 of the *CYP27A1* gene, confirming the diagnosis of cerebrotendinous xanthomatosis. This mutation has been previously reported in a few cases, all of which had associated clinical neurological abnormalities [1, 2]. In our case, however, there were no neurological symptoms. Therefore, a patient with tendon xanthomas should not be automatically diagnosed with familial hypercholesterolemia. Conversely, the absence of clinical neurological abnormalities should not exclude the diagnosis of cerebrotendinous xanthomatosis.
